# Topical Application of Tetrandrine Nanoemulsion Promotes the Expansion of CD4^+^Foxp3^+^ Regulatory T Cells and Alleviates Imiquimod-Induced Psoriasis in Mice

**DOI:** 10.3389/fimmu.2022.800283

**Published:** 2022-04-06

**Authors:** Shaokui Chen, Zibei Lin, Tianzhen He, Md Sahidul Islam, Long Xi, Ping Liao, Yang Yang, Ying Zheng, Xin Chen

**Affiliations:** ^1^State Key Laboratory of Quality Research in Chinese Medicine, Institute of Chinese Medical Sciences, University of Macau, Macau, Macau SAR, China; ^2^Department of Clinical Pharmacy, Guangzhou Hospital of Integrated Traditional and West Medicine, Guangzhou, China; ^3^Institute of Special Environmental Medicine, Nantong University, Nantong, China; ^4^MoE Frontiers Science Center for Precision Oncology, University of Macau, Macau, Macau SAR, China; ^5^Department of Pharmaceutical Sciences, Faculty of Health Sciences, University of Macau, Macau, Macau SAR, China

**Keywords:** psoriasis, tetrandrine, CD4^+^Foxp3^+^ regulatory T cells, TNFR2, nanoemulsion, Th17 cells

## Abstract

There is compelling evidence that CD4^+^Foxp3^+^ regulatory T cells (Tregs) are indispensable in the inhibition of autoimmune inflammatory responses, including psoriasis. Recently, we showed that systemically treatment with tetrandrine (TET), a two-pore channel inhibitor identified from the Chinese herb *Stephania tetrandra S*. Moor, could promote the proliferative expansion of Tregs in mice through stimulation of TNF-TNFR2 interaction. We thus hypothesized that topical administration of TET might also expand Tregs and consequently inhibit psoriasis. To this end, we developed a TET nanoemulsion and examined its effect on the expansion of Tregs after topical administration on mouse psoriasis induced by imiquimod. The result of our experiment showed that topical treatment with TET nanoemulsion markedly increased the proportion and number of Tregs in the spleen, as well as TNFR2 and Ki-67 expression by Tregs, in WT and TNFR1 KO mice, but not in TNFR2 KO mice. Consequently, TET nanoemulsion potently inhibited IL-17-expressing cells in the spleen and lymph nodes of imiquimod-treated WT mice, accompanied by decreased serum levels of IL-17A, INF-γ, and TNF and their mRNA levels in the flamed lesion. Importantly, TET nanoemulsion treatment markedly inhibited the development of psoriasis-like disease in WT and TNFR1 KO mice but not in TNFR2 KO mice. Therefore, our study indicates that the topical administration of TET could also stimulate the expansion of Tregs through the TNF-TNFR2 pathway. This effect of TET and its analogs may be useful in the treatment of inflammatory skin diseases such as psoriasis.

## Introduction

CD4^+^Foxp3^+^ regulatory T cells (Tregs) are crucial for maintaining immune homeostasis and preventing autoimmune responses (1). Therefore, targeting Tregs has become a strategy in treating major human diseases, such as allergic and autoimmune diseases, transplantation rejection, and GVHD (2). We, for the first time, found that tumor necrosis factor-alpha (TNF) through TNFR2, one of two receptors transducing TNF biological function, preferentially activate, expand, and maintain *in vivo* function and phenotype of Tregs (3, 4). Furthermore, we also found that the expression of TNFR2 identifies the maximally potent suppressive subset of human and mouse Tregs (5, 6). In contrast, Tregs without TNFR2 expression only had minimal or no suppressive activity (5, 7, 8). Therefore, targeting TNF-TNFR2 interaction represents a novel mechanism to stimulate Tregs for therapeutic purposes.

Tetrandrine (TET) is an immunosuppressive alkaloid isolated from the Chinese herb *Stephania tetrandra* (9). The previous study shows that TET can inhibit proinflammatory mediators such as TNF and nitric oxide produced by activated human monocytes or macrophages (10, 11). Recently, our group reported that TET potently inhibits the differentiation of proinflammatory Th1, Th2, and Th17 cells but spares the differentiation of Tregs (12). Furthermore, we have shown that TET inhibited the release of soluble TNF (sTNF) while enhanced transmembrane TNF (tmTNF) expression on antigen-presenting cells and consequently promotes the expansion of Tregs through tmTNF/TNFR2-dependent manner. This mechanism is attributable to the anti-inflammatory effect of TET in mouse colitis model (13). To therapeutically harness this property of TET while reducing potential toxicity caused by systemic delivery, we examined the effect of topical application of TET on skin inflammation. To this end, we developed a nanoemulsion (NE) system that enables hydrophobic TET to penetrate the skin. The result showed that topical administration of TET NE potently inhibited the mouse psoriasis-like disease induced by imiquimod (IMQ), which is associated with the expansion of Tregs and inhibition of inflammatory responses. Therefore, TET may be useful in treating human skin inflammatory diseases, and this idea merits further investigation.

## Material and Methods

### Mice and Reagents

Female 8-12 weeks old wild-type (WT) C57BL/6 mice, TNFR1^−/−^ or TNFR2^−/−^ mice with C57BL/6 background were used in this study. All mice were housed and treated with the approval of the Animal Research Ethics Committee of the University of Macau. PerCP-Cy5.5 anti-mouse TCRβ (clone: H57-597), PE anti-mouse IL-17A (clone: TC11-18H10), and PE anti-mouse CD120b/TNFR2 (clone: TR75-89) were purchased from BD Pharmingen (San Diego, CA). PE-Cy7 anti-mouse CD4 (clone: GK1.5), APC anti-mouse/rat Foxp3 (clone: FJK-16s), and FITC anti-human Ki-67 Monoclonal Antibody (clone: B56) were purchased from eBioscience. Hoechst 33342 was purchased from Invitrogen (Carlsbad, CA, USA). Anti-17 antibody (ab79056) and goat antirabbit IgG H&L (Alexa Fluor 488; ab150077) were purchased from Abcam, UK. TET (Cat: T2695) was purchased from Sigma-Aldrich. IMQ cream (5% w/w) was purchased from Health Care Limited (Loughborough, UK). Tacrolimus ointment (0.1% tacrolimus, Japan) was purchased from Astellas Pharma Tech Co., Ltd. Kolliphor^®^ HS 15 and Kollisolv^®^ PEG 400 were gift samples kindly offered by BASF Advanced Chemicals Co., Ltd, China. Labrafil M 1994 CS was a free sample from Gattefossé Co., Cedex, France. Milli-Q water was from a Millipore Direct-Q ultra-pure water system (Millipore, Bedford, USA). Carbopol 974N was purchased from Shanghai Chineway Pharmaceutical Technology Co., Ltd.

### Imiquimod Treatment

WT mice were randomly divided into 7 groups, and each group consisted of 9 mice. For induction of psoriasis-like disease, 3.125 mg of IMQ-containing cream was used to mice once daily for six consecutive days as previously reported (14). The mice groups were IMQ-only group, and others were topically smeared with 0.2 g high-concentration TET NE gel (9 mg/kg TET), medium-concentration TET NE gel (6 mg/kg TET), low-concentration TET NE gel (3 mg/kg TET), blank NE gel, and 0.3 mg of tacrolimus ointment (0.1% tacrolimus, Astellas Pharma Tech Co., Ltd., Japan) was treated after IMQ was given for 4 hours. The rest of one group was set as the untreated group.

For another experiment, WT, or TNFR1^−/−^ or TNFR2^−/−^, mice were randomly divided into three groups: untreated group, IMQ group, and IMQ+TET group, respectively. Each group consisted of 9 mice. 0.2 g of High-concentration TET NE gel was treated after IMQ was given for 4 hours.

### Scoring System

The Psoriasis Area and Severity Index (PASI) was used to score the severity of inflammation of the back skin including erythema, desquamation, and total PASI.

The score of erythema and desquamation ranging from 0 to 4 (0, none; 1, slight; 2, moderate; 3, marked; 4, very marked) and total PASI ranging from 0 to 8.

### Histopathology

All murine back skin samples were sectioned into 4-μm-thick slides and subsequently undergone hematoxylin-eosin (HE) staining according to the manufacturer’s instructions. The pathological changes per sample (magnification, ×100) was observed.

### RT-PCR

Total RNA was extracted from the skin samples using Trizol Reagent (Invitrogen). Messenger RNA (mRNA) expression levels was performed using SYBR Green system. GAPDH served as housekeeping gene. The relative mRNA expression was calculated using the comparative Ct method (ΔΔCt).

### ELISA

Mouse IFN-γ, TNF, and IL-17A were measured with the BD Cytometric Bead Array (CBA) Mouse Th1/Th2/Th17 Cytokine Kit (Cat#: BD 560485) by following the manufacturer’s instructions.

### Flow Cytometry and Intracellular Cytokine Staining

Single-cell suspension of splenic cells was collected in FACS buffer, fixed, and stained with the TCRβ PerCP-Cy5.5, CD4 PE-Cy7, Foxp3 APC, TNFR2 PE, and Ki-67 FITC.

For intracellular cytokines staining, cells were re-stimulated with phorbol myristate acetate (PMA, 20 ng/ml; Sigma-Aldrich St. Louis, MO) and ionomycin (1 μM; Sigma-Aldrich) in the presence of GolgiPlug (BD Biosciences) for 6 h. The cells were then fixed and permeabilized with Cytofix/Cytoperm (BD Pharmingen) and then stained with anti-IL-17A antibodies. All samples were measured with BD LSRFortessa. Data were analyzed using FlowJo software (Tree Star Inc., Ashland, OR).

### Immunofluorescence Staining

Mice skin was freshly excised into small pieces, washed three times in PBS, and embedded in a tissue freezing medium. 10 μm vertical sections were performed on Leica 230 CM1905 cryostat (Leica, Leica Biosystems, Buffalo Grove, IL). Then skin sections were fixed with 4% paraformaldehyde, blocked by 5% BSA and 0.3% Triton X-100 for 1 h, and with anti-IL17 (ab79056; 1:1000; Abcam) as primary antibody incubated overnight at 4°C, goat anti-rabbit IgG H&L (ab150077; 1:1000; Abcam) as secondary antibody. After that, sections were incubated with Hoechst 33342 (5 μg/ml) for 15 minutes and made into the slide for confocal laser scanning microscopy observation (Leica TCS SP8, Solms, Germany).

### Statistical Analysis

Statistical analysis was performed using Kruskal-Wallis test in SPSS (USA). Data are presented as mean ± SEM. *p* values < 0.05 were considered significant.

## Results

### Topic Administration With TET NE Upregulates tmTNF Expression by DCs and Expansion of TNFR2^+^ Tregs in IMQ-Treated Mice

**Figure 1:**
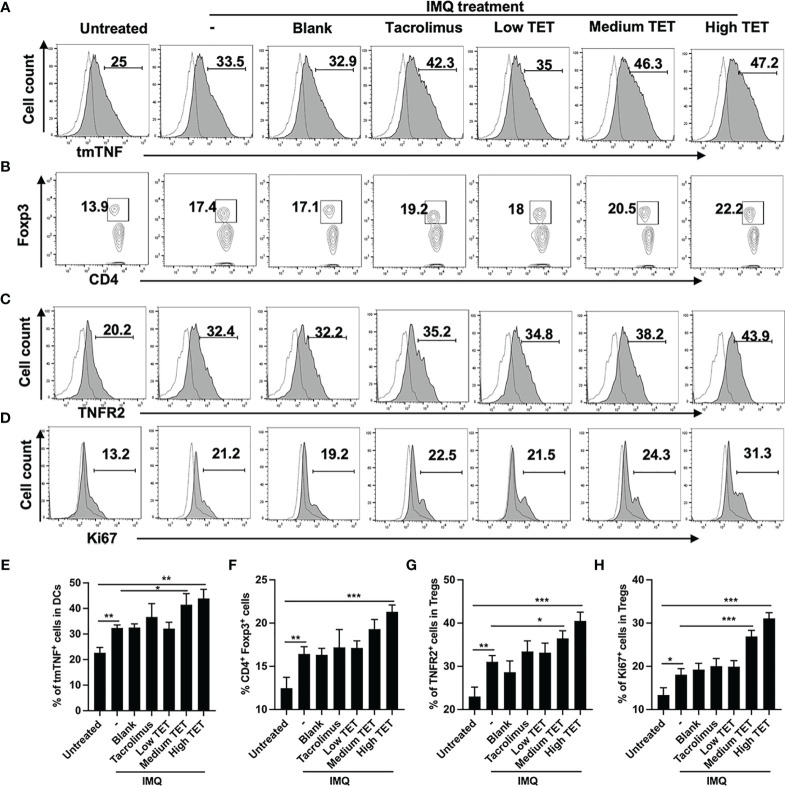
Topic administration with TET NE upregulates tmTNF expression by DCs and expansion of TNFR2^+^ Tregs in IMQ-treated mice. The C57BL/6 mice (N = 9) were treated with imiquimod (IMQ) for 6 days. TET NE treatment starts from the second day after IMQ treatment for 5 days. 24 hours later, the mice were sacrificed. tmTNF expression on CD11c^+^ cells **(A)**, Proportion of Tregs in CD4^+^ cells (gating on TCRβ^+^CD4^+^ cells, **B**), expression of TNFR2 by Tregs (gated on Foxp3^+^ Tregs, **C**) and expression of Ki-67 by Tregs (gated on Foxp3^+^ Tregs, **D**) in the spleen were analyzed by FACS. Dot histogram: isotype control. **(E–H)** show summarized data with statistical significance. Data were shown as means ± SEM. -: IMQ without treatment; Blank: IMQ + blank gel treatment. *P < 0.05, **P < 0.01, ***P < 0.001, compared with IMQ group (denoted as -).

To examine if TET NE treatment could up-regulate the expression of tmTNF on Dendritic cells (DCs) and consequently stimulate the proliferative expansion of Tregs in the psoriasis mouse model, WT C57BL/6 mice were treated with IMQ with or without low, medium, and high doses of TET NE (3, 6, 9 mg/kg, respectively). Tacrolimus, which was shown to be effective in treating psoriasis through either oral or topic administration (15), was used as the positive control. As shown in **Figures 1A, E**, after IMQ treatment, the expression of tmTNF on CD11c^+^ DCs in the spleen was increased by 34% (*P* < 0.001). The treatment with TET NE at 6, and 9 mg/kg per day further up-regulated tmTNF expression on CD11c^+^ DCs in WT mice by 85.2% and 88.8%, respectively (*P* < 0.001; **Figures 1A, E**). We also test related marker expression of tolerogenic DCs. The treatment with TET NE could down-regulated CD80 (**Figures S2A, C**) and MHC II (**Figures S2B, D**) expression on CD11c^+^ DCs in WT mice. The treatment with IMQ also increased the proportion of Treg cells in the splenic CD4 cells (**Figure 1B**, *P* < 0.01). The proportion of Foxp3^+^ cells in splenic CD4^+^ T cells was increased in a dose-dependent manner (17.8, and 28.2% increase in mice treated with TET NE at 6, and 9 mg/kg per day, respectively; **Figures 1B, F**; *P* < 0.05-0.001). Tregs retrieved from TET-NE treated psoriasis-like mice exhibited far more potent suppressive activity than Treg cells from blank gel treated group (**Figure S1**). Furthermore, TET NE induced the expansion of Tregs in the inflamed tissues (*P* < 0.05, **Figure S5**) and increased IL-10 levels in serum (*P* < 0.05, **Figure S6**). TNFR2 expression by Tregs from TET NE-treated WT mice was also markedly increased (*P* < 0.05; **Figures 1C, G**), indicative of highly suppressive phenotype (3). The expression of Ki-67 in Tregs was markedly increased after tetrandrine treatment (*P* < 0.05; **Figures 1D, H**). Ki-67 is a proliferating marker co-expressed with TNFR2 in highly suppressive Tregs (7). Therefore, topical treatment with TET NE could also stimulate the proliferative expansion of highly suppressive TNFR2^+^ Tregs.

### TET NE Reduces Inflammatory Responses in IMQ-Treated Mice

**Figure 2:**
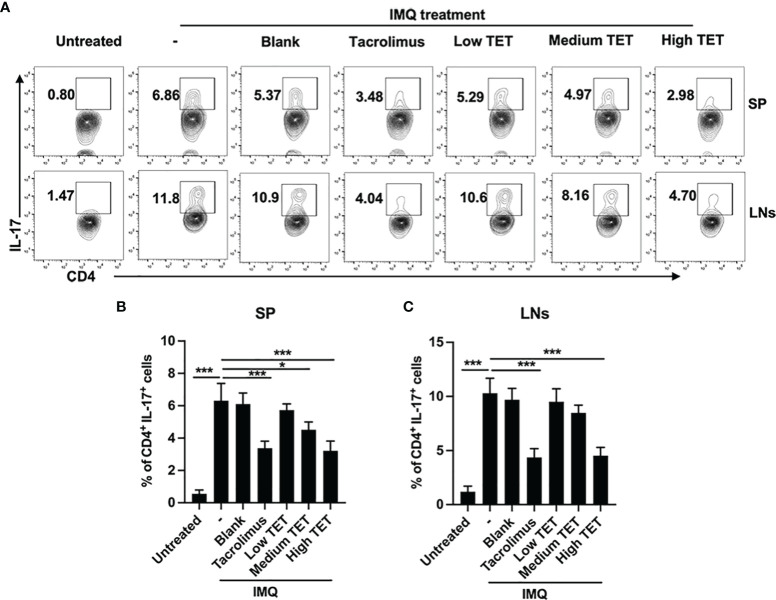
TET NE inhibits *in vivo* expression of IL-17 in IMQ-treated mice. The C57BL/6 mice (N = 9) were treated with IMQ for 6 days. TET NE treatment starts from the second day after IMQ treatment for 5 days. 24 hours later, the mice were sacrificed. Spleens and lymph nodes were harvested. The cells were re-stimulated with PMA and ionomycin in the presence of GolgiPlug for 6 h. Subsets of Th17 cells in CD4^+^ T cells gate were analyzed by intracellular staining of IL-17A in splenocytes from TET NE- or vehicle-treated psoriasis mice. FCM then analyzed the proportion of the Th17 subset in CD4^+^ T cells. **(A)** Representative FACS data are shown. The number in the FACS data indicates the proportion of gated cells. **(B, C)** show summarized data. Data were shown as means ± SEM. -: IMQ without treatment; Blank: IMQ + blank gel treatment. *P < 0.05, ***P < 0.001, compared with IMQ group (denoted as -).

**Figure 3:**
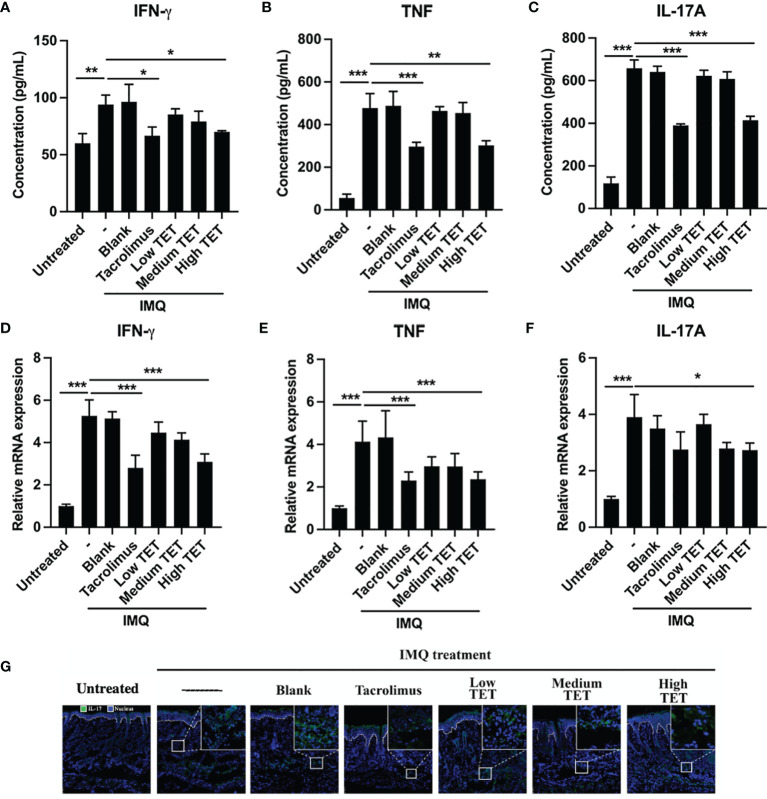
TET NE suppresses protein levels and mRNA expressions of pro-inflammatory cytokines in IMQ-treated psoriatic mice. WT mice (N = 9) were treated with IMQ for 6 days. TET NE treatment starts from the second day after IMQ treatment for 5 days. 24 hours later, the mice were sacrificed. The serum and skin were harvested. The protein level of IFN-γ **(A)**, TNF **(B)**, and IL-17A **(C)** in serum were measured by CBA kits. The mRNA expression of IFN-γ **(D)**, TNF **(E)**, and IL-17A **(F)** in skin lesions was measured by RT-PCR. **(G)** Immunofluorescence staining of IL-17A in the skin sections of mice in different groups. Data were shown as means ± SEM. -: IMQ without treatment; Blank: IMQ + blank gel treatment. *P < 0.05, **P < 0.01, ***P < 0.001, compared with IMQ group.

Pro-inflammatory IL-23/IL-17A axis plays a major role in the progression and perpetuation of psoriasis (16). Consistent with previous report (17), we were able to confirm that IMQ treatment resulted in a Th17 response, as evidenced by the marked increase of IL-17A-producing CD4 cells in the spleen (**Figures 2A, B**, *P* < 0.001) and LNs (**Figures 2A, C**, *P* < 0.001) (14). Treatment with TET NE could dose-dependently inhibit the proportion of IL-17-producing cells in IMQ-induced psoriasis mice (**Figures 2A–C**, *P* < 0.05-0.001). IMQ treatment also increased the IL-17A expression in CD4^-^ cells. However, treatment with TET NE could not inhibit the proportion of IL-17A-producing CD4^-^ cells in IMQ-induced psoriasis mice (**Figures S4A, B**). Furthermore, TET NE potently suppressed the IFN-γ, TNF, and IL-17A levels in serum (**Figures 3A–C**) and reduced their mRNA expression in the inflamed tissues in a dose-dependent manner (*P* < 0.05-0.001, **Figures 3D–F**). Notably, the inhibitory effect of a high dose of TET NE (9 mg/kg) on proinflammatory cytokines is comparable to that of tacrolimus (**Figures 2**, ** 3**). In addition, the immunofluorescent results indicated that the infiltration of IL-17^+^ cells in the dorsal skin of the IMQ-treated mice was markedly inhibited by the treatment with tacrolimus or TET NE (**Figure 3G**).

### TET NE Ameliorates Skin Lesion in IMQ-Induced Psoriasis Mice

**Figure 4:**
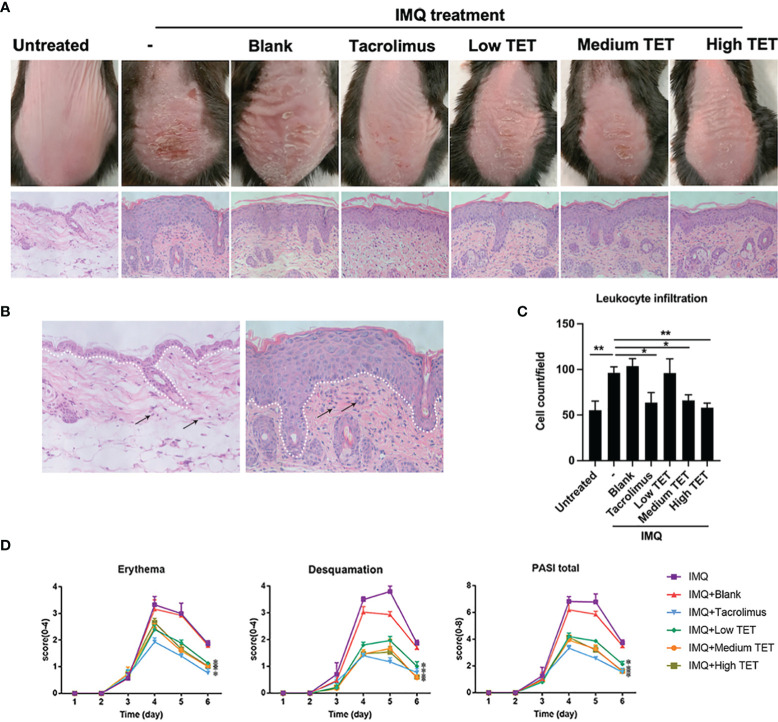
TET NE ameliorates skin lesion in IMQ-induced psoriasis mice. WT mice (N = 9) were treated with IMQ for 6 days. TET NE treatment starts from the second day after IMQ treatment for 5 days. 24 hours later, the mice were sacrificed. **(A)** Representative images of IMQ-induced psoriasis-like lesions and representative H&E staining of skin sections. **(B)** The white dotted line distinguished the epidermis (over the dotted line) from the dermis (below the dotted line). **(C)** Dermal inflammatory infiltrates on H&E staining (400×) were calculated with Image J software. **(D)** Clinical scores were assessed to monitor disease severity, including erythema, desquamation, and total PASI scores. Data were shown as means ± SEM. -: IMQ without treatment; Blank: IMQ + blank gel treatment. *P < 0.05, **P < 0.01, compared with IMQ group.

IMQ-induced skin inflammation has been widely used as a mouse psoriasis model (18, 19). Therefore, the effect of TET NE in IMQ-induced psoriasis-like skin inflammation was examined by simultaneous application of topical TET NE and IMQ application. The result showed that IMQ treatment induced inflamed appearance of mice dorsal skin (i.e., scaly plaques and redness, **Figures 4A, B**). This effect of IMQ was inhibited by the treatment with TET NE, and erythema and scaling on exposed skin was significantly inhibited by high dose of TET NE (9 mg/kg/day, **Figure 4D**, *P* < 0.01). The histopathological study showed that the increased thickness of skin epidermis induced by IMQ was almost completely reversed by the TET NE (**Figures 4A, B**). Leukocyte infiltration mirrors the kinetics of inflammatory cells in the inflammatory site (20, 21). As shown in **Figure 4C**, number of leukocytes in the skin was increased by two folds in mice treated with IMQ (*P* < 0.001), which was potently inhibited by TET in a dose-dependent manner (*P* < 0.05-0.01). The overall severity of disease of this model could quantitate by PASI (22). As shown in **Figure 4D**, PASI of IMQ-treated mice was markedly lower by the treatment with TET NE at medium and high doses, and the therapeutic effect of TET NE was comparable to that of Tacrolimus.

### TET NE Reduces IMQ-Induced Psoriasis-Like Skin Inflammation *via* TNFR2

**Figure 5:**
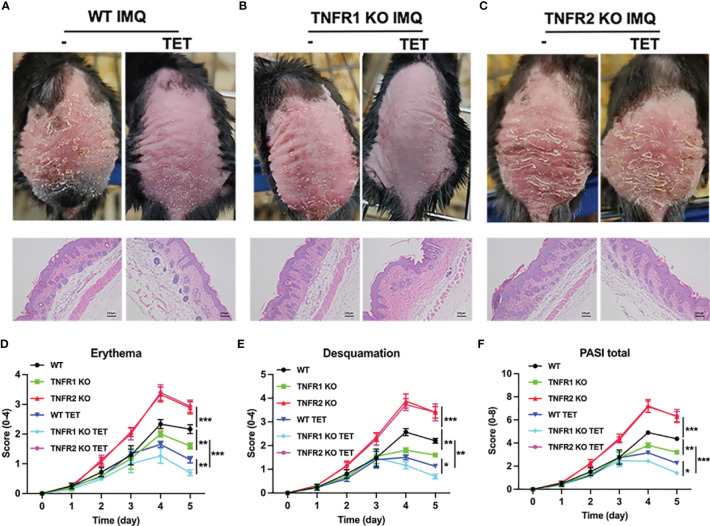
TET NE reduces IMQ-induced psoriasis-like skin inflammation *via* TNFR2. WT, or TNFR1^-/-^ or TNFR2^-/-^ mice (N = 9) were treated with IMQ for 6 days. TET NE treatment starts from the second day after IMQ treatment for 5 days. 24 hours later, the mice were sacrificed. **(A–C)** Representative images of IMQ-induced psoriasis-like lesions and representative H&E staining of skin sections. **(D–F)** Clinical scores were assessed to monitor disease severity, including erythema **(D)**, desquamation **(E)**, and total PASI scores **(F)**. Data were shown as means ± SEM. *P < 0.05 **P < 0.01, ***P < 0.001.

Previously, we showed that TET inhibited colitis through Tregs in TNFR2 dependent manner (13). To assess if this amelioration from psoriasis-like skin inflammation after TET NE treatment (**Figure 4**) was attributed to TNFR2 expression, WT, or TNFR1^-/-^ or TNFR2^-/-^ mice were treated with IMQ daily for 6 days. From the second day of IMQ application, some mice were also treated with TET NE (9 mg/kg/day) simultaneously for five consecutive days. Consistent with our previous report that IMQ treatment resulted in a more severe skin inflammation in TNFR1 KO mice and a milder disease in TNFR1 KO mice, as compared to that in WT mice (**Figures 5A–F**) (17). TET NE treatment markedly inhibited the skin inflammation (i.e., reduction in erythema, desquamation, PASI score, and epidermal thickness) induced by IMQ in WT and TNFR1^-/-^ mice (**Figures 5A–F**). In sharp contrast, TET NE treatment failed to affect the severity of IMQ-induced psoriasis-like skin inflammation in TNFR2^-/-^ mice (**Figures 5A–F**).

### The Effect of TET NE Is Mediated by the Expansion of Naturally Occurring CD4^+^ Foxp3^+^ Tregs *via* TNF-TNFR2 Signaling

**Figure 6:**
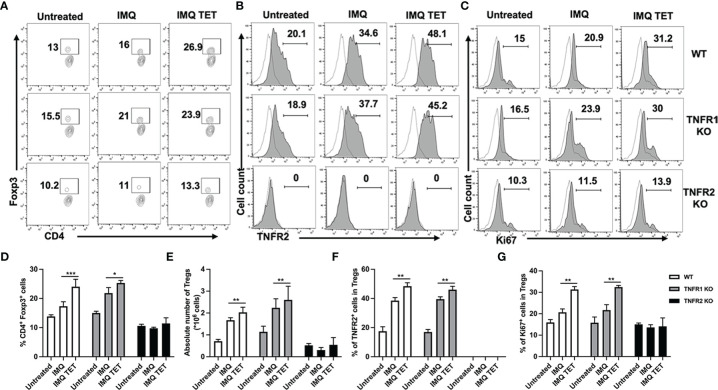
The anti-inflammatory effect of TET NE is based on the expansion of naturally occurring CD4^+^ Foxp3^+^ regulatory T cells by TNF-TNFR2 signaling. WT, or TNFR1^-/-^ or TNFR2^-/-^ mice (N = 9) were treated with IMQ for 6 days. TET NE treatment starts from the second day after IMQ treatment for 5 days. 24 hours later, the mice were sacrificed. The proportion of Tregs in CD4 cells (gating on TCRβ^+^CD4^+^ cells, **A, D**), expression of Ki-67 by Tregs (gated on Foxp3^+^ Tregs, **C, G**), and expression of TNFR2 by Tregs (gated on Foxp3^+^ Tregs. **B, F**) in the spleen were analyzed with FACS. The number of Tregs in the spleen is shown in **(E)**. **(A–C)** Representative FACS data are shown. The number in the FACS data indicates the proportion of gated cells. Dot histogram: isotype control. **(D–G)** show summarized data. Data were shown as means ± SEM. *P < 0.05, **P < 0.01, ***P < 0.001, compared with IMQ group.

We next examined even if the expansion of Tregs in IMQ-induce psoriasis mice after TET NE treatment (section 3.1) is attributed to TNF-TNFR2 signaling. As described in the previous section, we simultaneously treated WT or TNFR1^-/-^ or TNFR2^-/-^ mice with IMQ and TET NE (9 mg/kg/Day). On day 6, mice were sacrificed, and the number and phenotype of Tregs in spleen and lymph nodes at axillary, inguinal, and mesenteric regions were analyzed. Strikingly, after TET NE treatment, the proportion of splenic Tregs (~1.15-1.68 (approx.) fold; *P* < 0.01; **Figures 6A, D**) markedly increased in WT and TNFR1^-/-^ mice in comparison with the vehicle group. The absolute number of Tregs in the spleen was also increased after TET NE treatment in both WT and TNFR1^-/-^ mice (*P* < 0.01; **Figure 6E**). In contrast, the proportion and the absolute number of splenic Tregs remained unaltered (*P* > 0.05; **Figures 6A, D, E**). A similar phenomenal observation was found in lymph nodes (auxiliary and inguinal regions) after TET NE treatment in those mice groups (data not shown). At the same time, the TNFR2 expression on Tregs (*P* < 0.01; **Figures 6B, F**) and Ki67 expression on Tregs (*P* < 0.05; **Figures 6C, G**) in splenic CD4^+^ cells were markedly increased after TET NE treatment in WT and TNFR1^-/-^ mice. In sharp contrast, TET NE treatment failed to induce the expression Ki-67 by Tregs (*P* > 0.05; **Figures 6C, G**) in TNFR2^-/-^ mice. These data indicate that TNF-TNFR2 interaction is required for the expansion of Tregs in TET NE-treated mice.

### TET NE Suppresses mRNA and Protein Expressions of Pro-Inflammatory Cytokines in TNFR2 Dependent Manner

**Figure 7:**
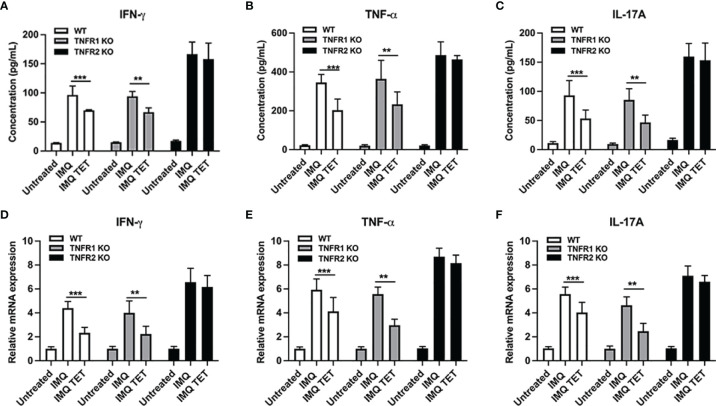
TET NE suppresses mRNA and protein expressions of pro-inflammatory cytokines in TNFR2 dependent manner. WT, or TNFR1^-/-^ or TNFR2^-/-^ mice (N = 9) were treated with IMQ for 6 days; TET NE treatment starts from the second day after IMQ treatment for 5 days. 24 hours later, the mice were sacrificed. Skin lesion samples were rapidly frozen in liquid nitrogen and stored at -80°C. The protein level of IFN-γ **(A)**, TNF **(B)**, and IL-17A **(C)** in serum were measured by CBA kits. The mRNA expression of IFN-γ **(D)**, TNF **(E)**, and IL-17A **(F)** in skin lesions was measured by RT-PCR. Data were shown as means ± SEM. **P < 0.01, ***P < 0.001, compared with IMQ group.

Effects of TET NE on pro-inflammatory cytokine expression were also observed *via* flow cytometry and RT-PCR after TET NE treatment. As shown in **Figures 7A–C**, pro-inflammatory cytokine expression in serum was increased by treatment with IMQ in WT, TNFR1^-/-^ and TNFR2^-/-^ mice. Similarly, the mRNA expressions of pro-inflammatory cytokine in the skin were increased in WT, TNFR1^-/-^ and TNFR2^-/-^ mice (**Figures 7D–F**). Compared with vehicle control mice, these cytokine levels were decreased in both WT and TNFR1^−/−^ mice (*P* < 0.01; **Figures 7A–C**) while remained unaltered in TNFR2^−/−^ mice after treatment with TET NE. IFN-γ, TNF, and IL-17 expression were down-regulated after treatment with TET NE in WT mice and TNFR1^-/-^ mice (**Figures 7A–F**). Meanwhile, pro-inflammatory cytokine expression showed no significant change after treatment with TET NE in TNFR2^-/-^ mice (**Figures 7A–F**). These data indicate that TET NE inhibits the expressions of pro-inflammatory cytokines in TNFR2 dependent manner.

## Discussion

Impaired Tregs function is considered a potential factor for the caused skin inflammation in psoriasis (23). Accordingly, Tregs from the blood of psoriatic patients is impaired in their capacity to inhibit effector T cells (Teff cells) *in vitro* (23) and lesioned skin of psoriatic patients exhibits an abnormally low Treg/Teff cell ratio (24). Thus, boosting the Tregs function in psoriasis patients has the potential to alleviate psoriasis. Strikingly, our previous work has demonstrated that TET, a traditional Chinese medicine-derived small compound could promote Tregs expansion by enhancing tmTNF expression on antigen-presenting cells through tmTNF/TNFR2 signaling *in vivo* (13). Simultaneously, TET did not promote or inhibit the *in vitro* generation of iTregs (12). Our results showed that TET NE could increase the proportion of Foxp3^+^ cells in skin CD4^+^ T cells than Blank gel. In another recent study, we found that, the expression of genes related to ECM-receptor interaction, nitrogen metabolism, and pyruvate metabolism was significantly down-regulated in TNFR2^-^ Tregs. These data support the notion that TNFR2 expression on Tregs is crucial for their proliferative expansion and consequently dampening inflammatory responses in IMQ-treated mice (17). Thus, it is highly likely that the reduced number of Tregs in TNFR2^-/-^ mice is responsible for the exaggerated inflammation in IMQ-treated mice. This idea is further supported by our observation that adoptive transfer of WT Tregs largely blunted the exaggerated inflammation in IMQ-treated TNFR2^-/-^ mice, and down-regulation of proliferation-related genes in Tregs deficient in TNFR2.

TET has a molecular weight of 622.750, and its log P is 3.55, which indicates that transdermal delivery of it would be a challenge. Numerous studies have demonstrated that NE can be a high-potential transdermal vehicle (25) and exhibits a better anti-psoriatic activity (26) when as carrier to deliver drug in treatment of psoriasis. NE is a homogeneous and transparent solution consists of oil, surfactant, cosurfactant and water at a specific ratio, which usually serves as a carrier for insoluble, hydrophobic, or hydrolysable pharmaceutical ingredients. Moreover, high and low energy emulsification methods both can produce NE, thermo-kinetically and thero-dynamically (27). The latter technique is more energy-saving and simple to be carried out, which ensures industrial large-scale production of NE. Here we developed a TET NE formulation *via* low energy emulsification methods for treating psoriasis, which is of great significance to development of a practical anti-psoriatic agent. Thus, we developed topical TET NE to surpass this poor solubility of TET and make any easy option to excel its biodistribution in skin inflammatory site in psoriasis patients. Our study strongly provided evidence that TET NE can protect the immune response in IMQ-induced psoriasis mice *via* promoting Tregs expansion. Especially high concentration of TET NE was able to mimic the same pattern in the alleviation of psoriasis severity provided by tacrolimus ointment, a commercially available drug for psoriasis treatment.

Our group reported that TET potently inhibits the differentiation of proinflammatory Th1, Th2, and Th17 cells but spares the differentiation of Tregs (12). Furthermore, we have shown that TET inhibited the release of sTNF while enhanced tmTNF expression on antigen-presenting cells and consequently promotes the expansion of Tregs through tmTNF/TNFR2-dependent manner. This mechanism is attributable to the anti-inflammatory effect of TET in mouse colitis model (13). More recently, it was reported that the inhibition of differentiation of proinflammatory T helper cells may also contribute to the anti-inflammatory effect of TET. For example, it was reported that TET, by activating aryl hydrocarbon receptor (AHR), inhibited the generation of Th17 cells and promoted the conversion of naïve CD4 T cells into induced Tregs (iTreg), and consequently suppressed the inflammatory responses in mouse collagen-induced arthritis (28, 29). Therefore, it is likely that promotion of Tregs expansion and inhibition of proinflammatory subsets of Th cells cooperatively contribute to the anti-psoriatic effect of TET NE found in this study. Further, IL-23/IL-17 axis plays a central role in developing various autoimmune diseases, including human psoriasis and IMQ-induced murine psoriasis (14). It has demonstrated that Th17 cells were increased in psoriatic plaques and the peripheral blood of patients with psoriasis and correlated with disease severity (30). Therefore, searching for specific agents targeting these subsets has clinical significance. Strikingly, in our study, TET NE can also drastically reduce the levels and expression of IL-17 and the infiltration of Th17 cells at inflammation site that confers TET NE can significantly inhibit the IL-23/IL-17 axis in IMQ-induced psoriasis mice. These results are in concordance with our observations that TET inhibits differentiation of proinflammatory Th17 cells (12). A similar statement was demonstrated in a recent study of TET as an activator aryl hydrocarbon receptor that can inhibit the generation of Th17 cells and consequently suppressed the inflammatory responses in mouse collagen-induced arthritis (28, 29). Increasing evidence has demonstrated that aberrantly activated γδT cells may direct the pathogenesis of autoimmune disorders, such as psoriasis (31). Previous studies have reported that, in IMQ-induced mouse model of psoriasis-like disease, γδT cells were producing more IL-17A compared with control group (31, 32). We were able to confirm the result of these previous reports. Nevertheless, TET had no effects on the ratio of IL-17A expressed CD4^-^ γδT cells. Multiple immune effector cells, including T cells, B cells, macrophages, dendritic cells, NK cells, neutrophils, eosinophils, and mast cells, express IL-10 which plays an immunosuppressive function (33). It was also reported that anti-inflammatory IL-10 endowed Treg cells with the ability to suppress pathogenic Th17 cell responses (34). Our results showed that TET NE could increase the expression of IL-10 in serum than Blank gel. More importantly, we have deciphered that these reductions in the levels and expression of pro-inflammatory cytokine response and expansion of Tregs by TET NE in IMQ-induced are prominently mediated by TNF/TNFR2 signaling axis. However, the systemic effect of TET NE on the expansion of Tregs and the function of other immune cells, should be further investigated.

Certain types of DCs, such tolerogenic DCs, have the capacity to induce Tregs by expressing immunosuppressive molecules (35). Tolerogenic DCs often display an immature or semi-mature phenotype that is characterized by low expression of CD80 and MHC molecules and altered cytokine production (36). As shown in our study, DCs from TET-treated mice acquired the capacity to stimulate the expansion of Tregs. Moreover, although the pathogenesis of psoriasis depends on the activation of T cells, innate immune cells also play important roles in disease progression. Among them, DCs as professional antigen-presenting cells have a potent capacity to prime naïve T cells and activate autoreactive response (22). We previously reported that DC-mediated delivery of nanoparticle-coated drugs provided a promising option in treating psoriasis (37, 38). At the same time, our previous study suggested that DCs treated with TET acquired the capacity to stimulate the expansion of Tregs through up-regulation of tmTNF on their surface (13). Indeed, our developed TET NE markedly increased the percentage of tmTNF expression on DCs as well and consequently induced the proliferative expansion of highly suppressive Tregs in the IMQ-induced psoriasis model. Therefore, it would be an exciting direction to address in the future that TET NE may be more useful in generating DC-based cellular therapy for psoriasis.

Taken together, our data indicate that TET NE could enhance tmTNF expression on DCs, to promote the proliferative expansion of highly immunosuppressive Tregs, and to inhibit IL-17/IL-23 axis through the TNF-TNFR2 interaction. Thus, these properties of TET NE may be therapeutically harnessed in the treatment of psoriasis. Moreover, as the pathogenesis of autoimmune diseases often involves a complicated network of various factors, compounds from traditional Chinese medicine such as TET, which exhibit multiple influences against autoimmune inflammation, might need more attention.

## Data Availability Statement

The original contributions presented in the study are included in the article/**Supplementary Material**. Further inquiries can be directed to the corresponding authors.

## Ethics Statement

The animal study was reviewed and approved by Animal Research Ethics Committee of the University of Macau.

## Author Contributions

SC, ZL and LX performed the experiments. SC, ZL, TH, MI, PL, YY, YZ, and XC designed the experiments and wrote the manuscript. All authors contributed to the article and approved the submitted version.

## Funding

This work was funded by grants from Macau Science and Technology Development Fund (0099/2021/A2, 0086/2021/A2 and 0056/2019/AFJ), Applied Research Programs of Guangdong-Hong Kong-Macao Innovation Center sponsored by Guangzhou Development District (EF032/ICMS-CX/2021/RITH) and the Science and Technology Development Fund, Macau SAR (SKL-QRCM(UM)-2020-2022).

## Conflict of Interest

The authors declare that the research was conducted in the absence of any commercial or financial relationships that could be construed as a potential conflict of interest.

## Publisher’s Note

All claims expressed in this article are solely those of the authors and do not necessarily represent those of their affiliated organizations, or those of the publisher, the editors and the reviewers. Any product that may be evaluated in this article, or claim that may be made by its manufacturer, is not guaranteed or endorsed by the publisher.
