# Supplementary material for: Topical Application of Tetrandrine Nanoemulsion Promotes the Expansion of CD4+Foxp3+ Regulatory T Cells and Alleviates Imiquimod-Induced Psoriasis in Mice
Source: Front Immunol. 2022 Apr 6;13:800283. doi: 10.3389/fimmu.2022.800283 (PMC9020220; doi:10.3389/fimmu.2022.800283)
Supplement: Supplementary file 1 [file Presentation_1.pdf]

Supplementary materials

Topical application of tetrandrine nanoemulsion promotes the expansion of $CD4^+Foxp3^+$ Regulatory T Cells and alleviates imiquimod-induced psoriasis in mice

Shaokui Chen[1†], Zibei Lin[1,2†], Tianzhen He[1,3], Md Sahidul Islam[1], Long Xi[1], Ping Liao[1], Yang Yang[1], Ying Zheng[1,5*], and Xin Chen[1,4,5*]

1. State Key Laboratory of Quality Research in Chinese Medicine, Institute of Chinese Medical Sciences, University of Macau, Macau SAR 999078, China

2. Department of Clinical Pharmacy, Guangzhou Hospital of Integrated Traditional and West Medicine, Guangzhou 510000, China

3. Institute of Special Environmental Medicine, Nantong University, Nantong 226000, China

4. Department of Pharmaceutical Sciences, Faculty of Health Sciences, University of Macau, Macau SAR 999078, China

5. MoE Frontiers Science Center for Precision Oncology, University of Macau, Macau SAR 999078, China

[†]These authors have contributed equally to this work.

* Corresponding author: Dr. Xin Chen, Institute of Chinese Medical Sciences, University of Macau, Avenida da Universidade, Taipa, Macau SAR 999078, China.Tel.: (853) 8822 4513; Fax: (853) 2884 1358. E-mail address: xchen@umac.mo

Dr. Ying Zheng, Institute of Chinese Medical Sciences, University of Macau, Avenida da Universidade, Taipa, Macau SAR 999078. E-mail address: yzheng@umac.mo

Supplementary Figure

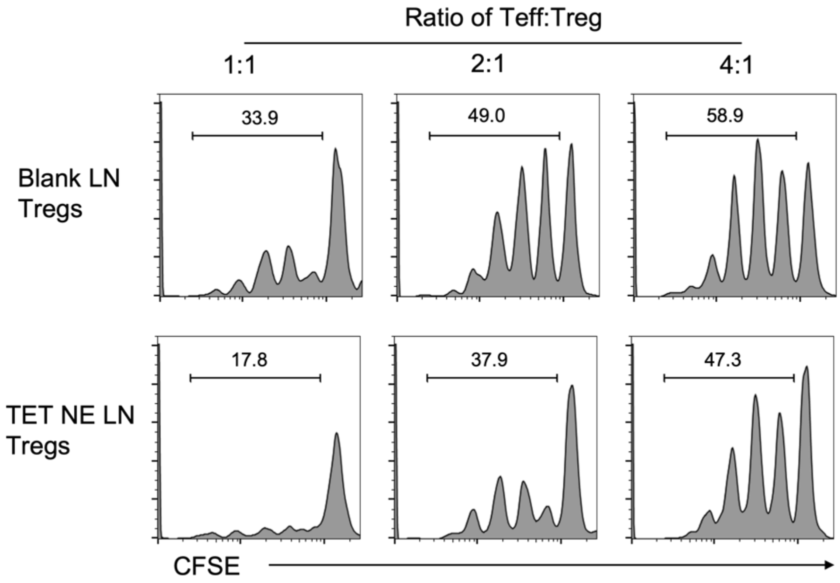

Figure S1. Highly suppressive Tregs accumulated in the TET NE treated psoriatic mice. Flow-sorted $CD4^+CD25^+$ Tregs or $CD4^+CD25^-$ T cells ($5*10^4$ cells/well) were labeled with CFSE and cultured alone or cocultured with the indicated numbers or ratios of flow-sorted CD4 subsets from spleen and LNs of TET NE and Blank NE-treated mice. The percentages of CFSE-diluted cells are shown in the histograms. Figure show a representative data of at least three separate experiments with similar results.

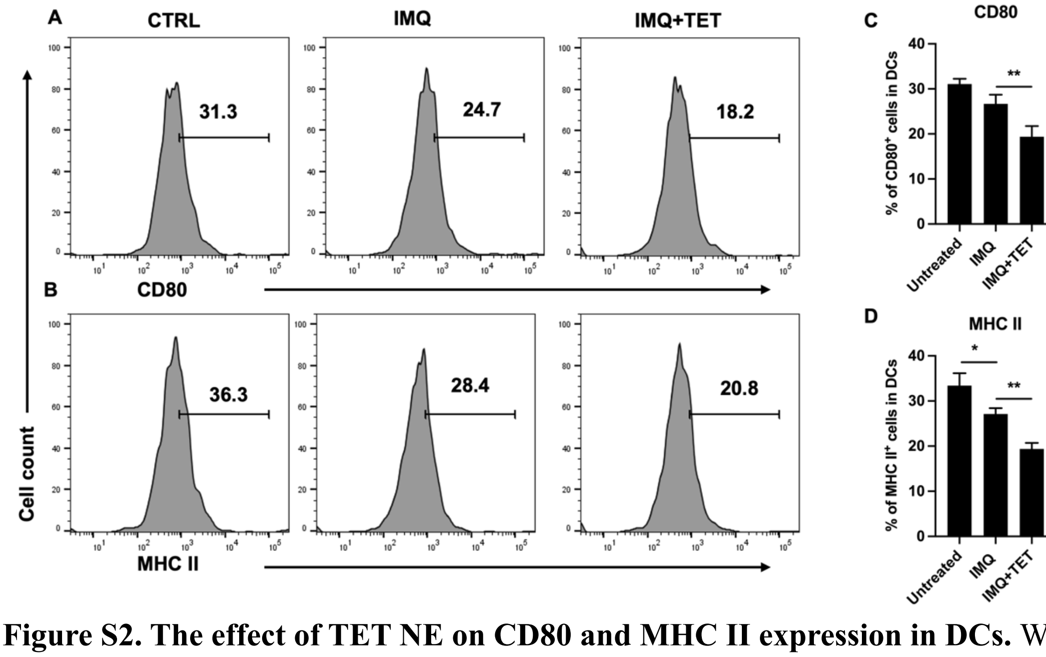

Figure S2. The effect of TET NE on CD80 and MHC II expression in DCs. WT mice (N = 5) were treated with IMQ for 6 days. TET NE treatment starts from the second day after IMQ treatment for 5 days. 24 hours later, the mice were sacrificed.

CD80 expression on CD11c$^+$ cells (A) and MHC II expression on CD11c$^+$ cells (B) in the spleen were analyzed by FACS. (C and D) show summarized data with statistical significance. Data were shown as means ± SEM. * $P < 0.05$, ** $P < 0.01$, compared with IMQ group.

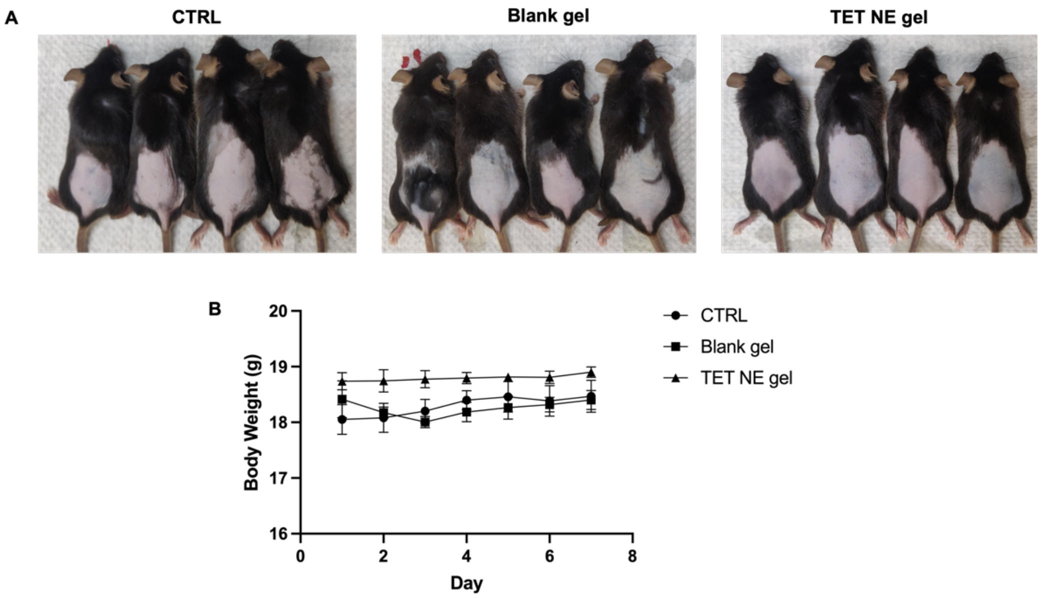

Figure S3. Skin toxicity of TET NE for multiple application. The C57BL/6 mice (N = 4) were treated with Blank gel and TET NE gel for 6 days. (A) Representative images of skin lesions. (B) Body weight.

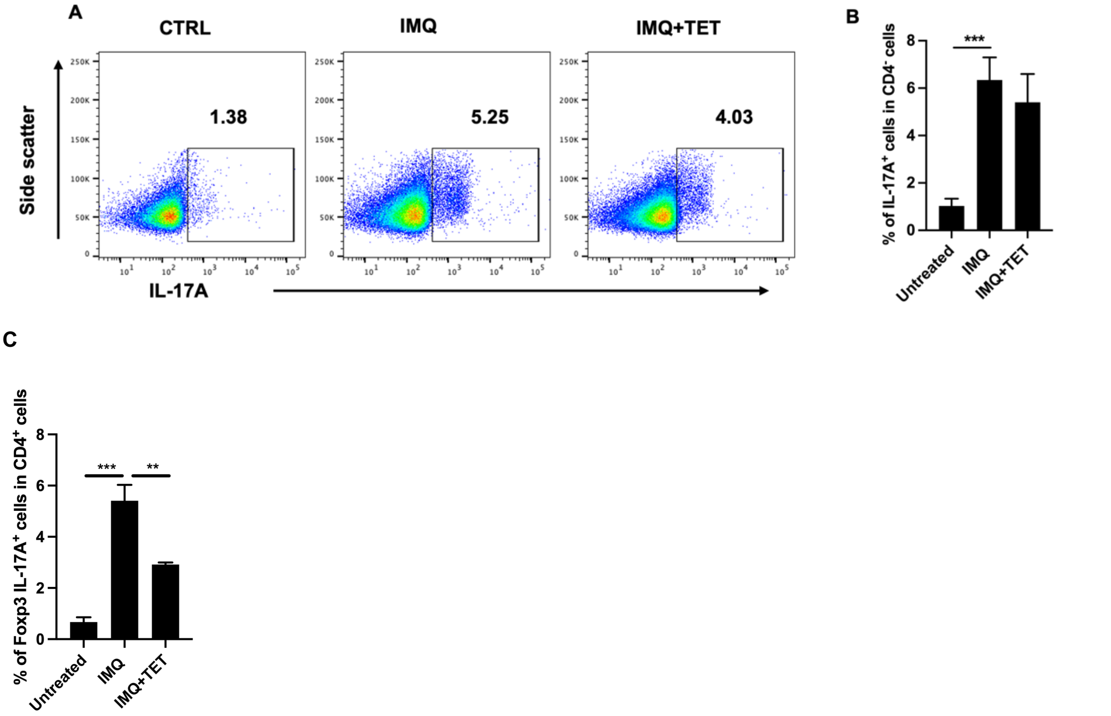

Figure S4. The effect of TET NE on IL-17A expression in CD4 cells. The C57BL/6 mice (N = 5) were treated with IMQ for 6 days. TET NE treatment starts from the

second day after IMQ treatment for 5 days. 24 hours later, the mice were sacrificed. Spleens and lymph nodes were harvested. The cells were re-stimulated with PMA and ionomycin in the presence of GolgiPlug for 6 h. IL-17A expression in $CD4^-$ T cells gate was analyzed by intracellular staining of IL-17A in splenocytes from TET NE- or vehicle-treated psoriasis mice. FCM then analyzed the proportion of the Th17 subset in CD4 T cells. (A) Representative FACS data are shown. The number in the FACS data indicates the proportion of gated cells. (B) show summarized data. (C) show IL-17 vs Foxp3 in CD4 positive cells. Data were shown as means ± SEM. ** $P < 0.01$, *** $P < 0.001$, compared with IMQ group.

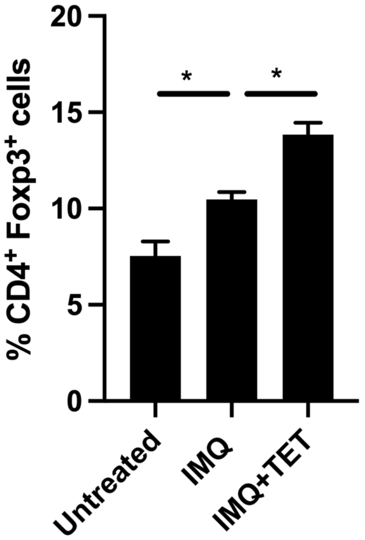

Figure S5. The effect of TET NE on expansion of Tregs in skin. WT mice (N = 5) were treated with IMQ for 6 days. TET NE treatment starts from the second day after IMQ treatment for 5 days. 24 hours later, the mice were sacrificed. The skin was harvested. The expansion of Tregs in skin lesions was measured by flow. Data were shown as means ± SEM. * $P < 0.05$, compared with IMQ group.

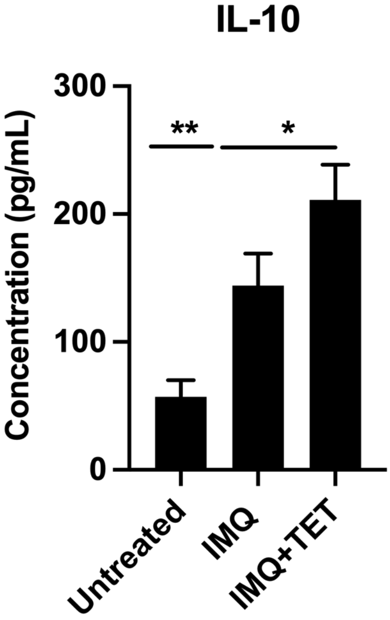

Figure S6. The effect of TET NE on IL-10 expression in serum. WT mice (N = 5) were treated with IMQ for 6 days. TET NE treatment starts from the second day after IMQ treatment for 5 days. 24 hours later, the mice were sacrificed. The serum was harvested. The protein level of IL-10 in serum were measured by CBA kits. Data were shown as means ± SEM. * $P < 0.05$, ** $P < 0.01$, compared with IMQ group.

A **B**

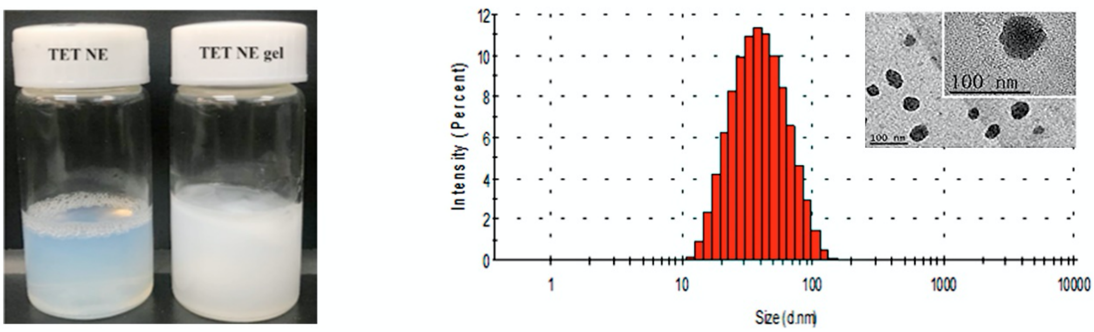

Figure S7. Appearance and size distribution of TET NE. (A) The left one was TET NE and right one is TET NE gel. Labrafil M 1944 CS (10%, v/v), Solutol HS 15 (10%, v/v) and PEG 400 (5%, v/v) were weighed into a vial and mixed homogeneously. TET was then added and dissolved with constantly stirring by a magnetic stirring bar at a 40 °C waterbath. Afterwards, diluted water (75%, v/v) was added drop by drop accompanied with a 15-minute stirring and transparent TET NE was formed. Carbopol 974N was selected as the loading matrix of TET NE. 2% (w/v) Carbopol 974N was well swelling in diluted water with overnight constant stirring to form a sticky watery gel. TET NE of different ratio was drop-wise added into the gel then neutralized to pH 7 by adding triethanolamine. Gels with a TET concentration of 900, 600 and 300 μg/g·gel was respectively manufactured for subsequent animal experiment as high TET gel, medium TET gel and low TET gel. Blank NE gel (Blank NE gel) was prepared with mere blank NE (without TET). (B) Red curve exhibits PDI of TET NE. Prepared TET NE was diluted in Milli-Q water and its particle size, polydispersity index (PDI) and zeta potential of NE were measured using a Malvern Zetasizer Nano-ZS system (Malvern Instruments, UK). Triplicated measurements were conducted for each sample. TET NE were diluted by water up to 50 times and dropped onto surface of a specific copper mesh for 3 mins then wiped away. Subsequently the meshes were covered by a drop of 3% phosphotungstic acid, which stayed for 3mins then was wiped. Finally, prepared samples were photographed using a transmission electron microscope (TEM).

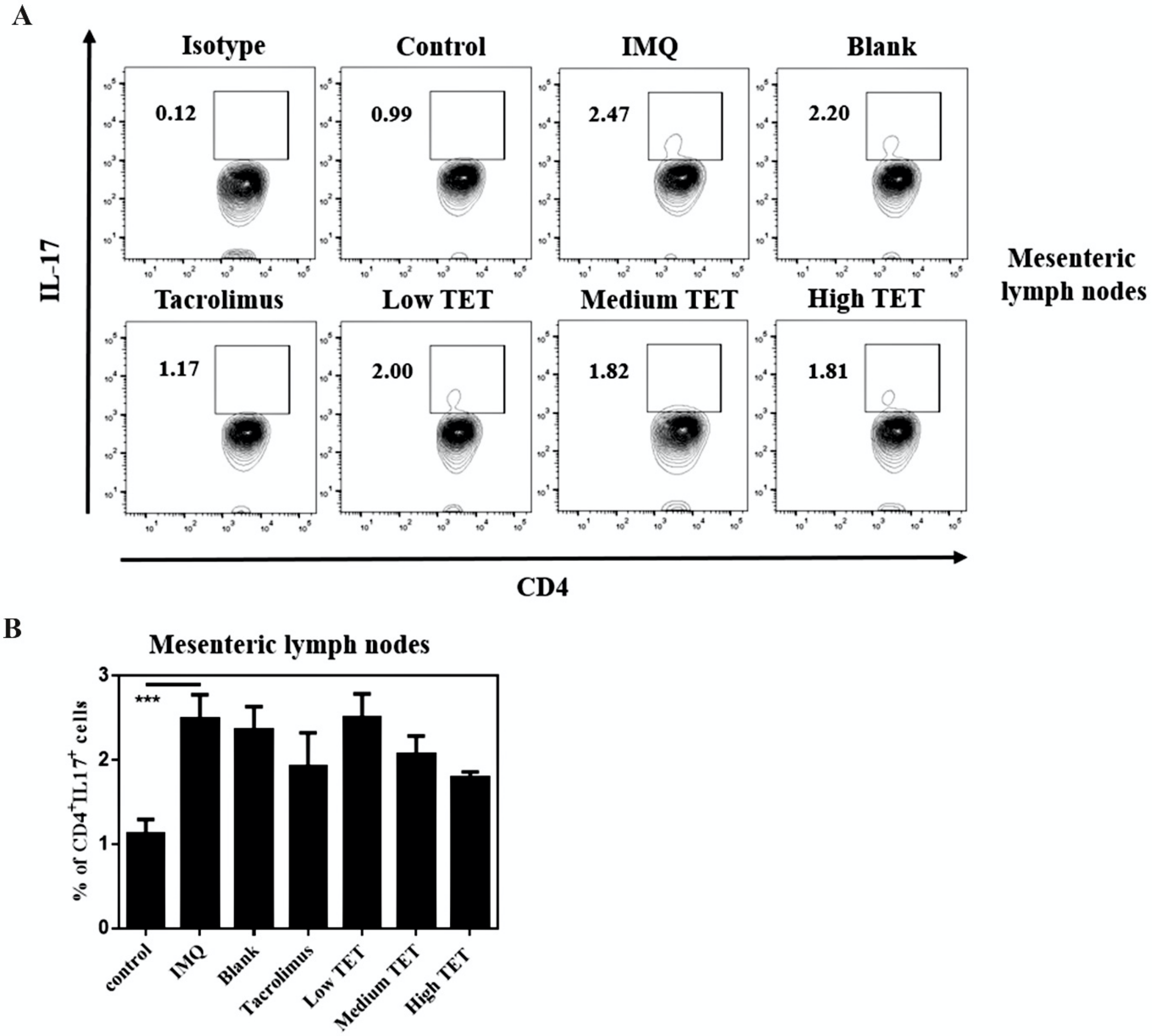

Figure S8. *In vivo* expression of IL-17 in mesenteric lymph nodes of IMQ-treated mice. Expression of cytokine IL-17 was analyzed on gated CD4 cells, performing by FACS. FACS results data are shown in (A). Number in the FACS data indicates the proportion of gated cells. (B) exhibited summarized data (means ± SEM). ***p<0.001, as compared with control group.

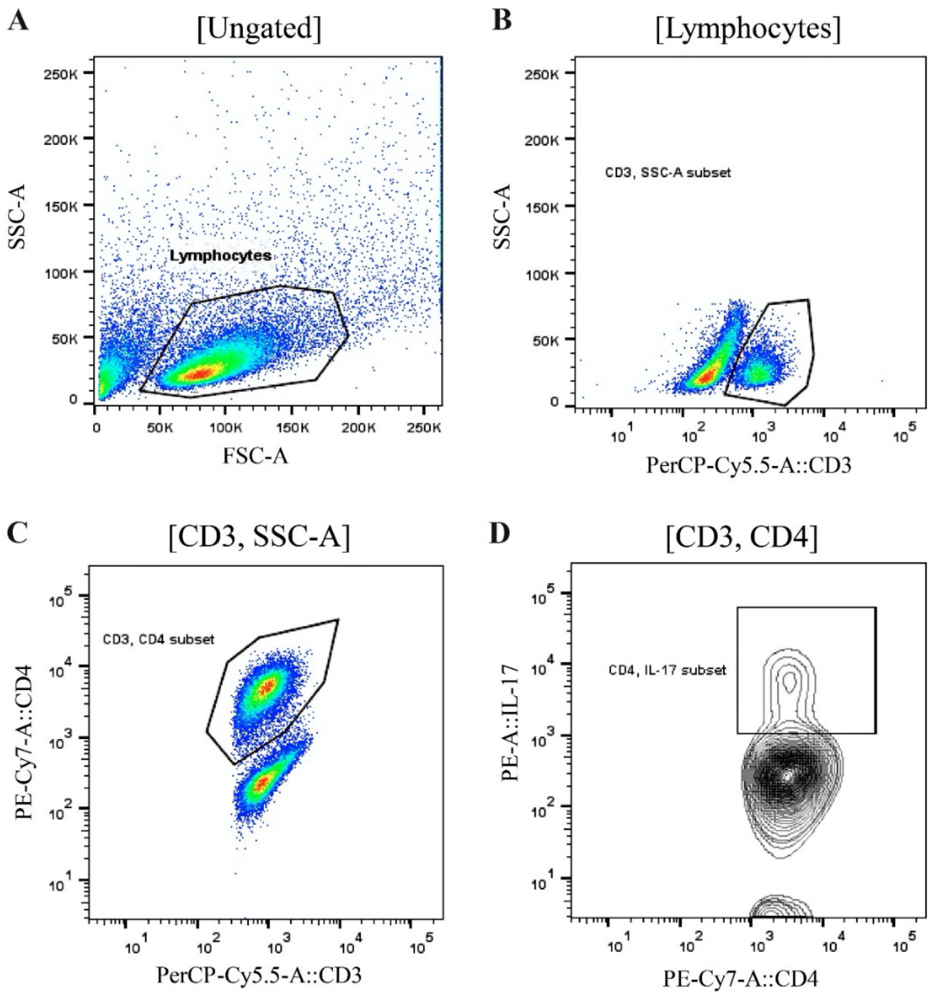

Figure S9. Gating strategy of IL-17. Tissues like lymph nodes and spleens were harvested and grinded into cells, then cells were stained with labeled antibodies, and expression data were collected by BD Fortessa and analyzed by using Flow Jo. (A) Lymphocytes were gated based on FSC and SSC, followed by a $CD3^+$ cell gate (B). $CD4^+$ subset (C) was gating in the $CD3^+$ cells, then IL-17 (D) was gating out in the $CD3^+$ $CD4^+$ subset.

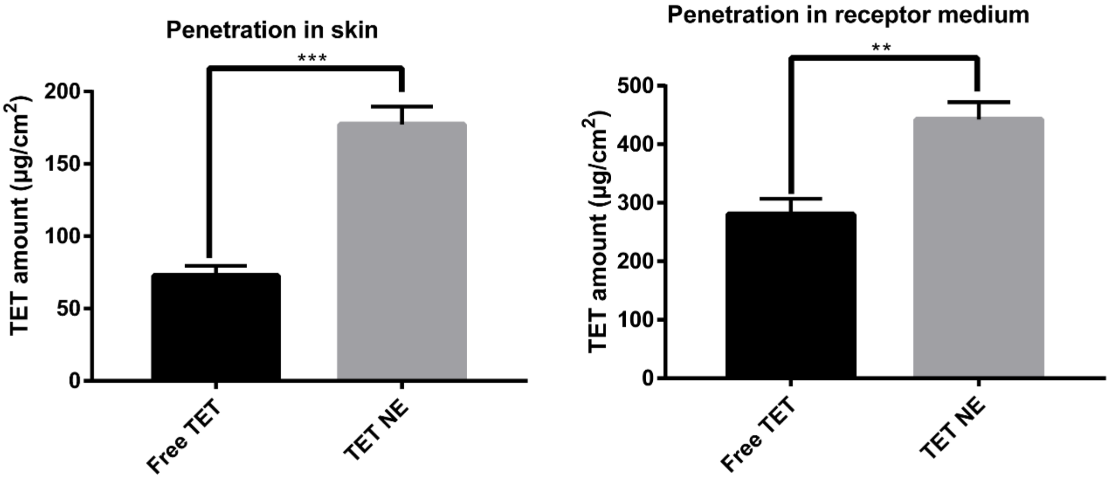

Figure S10. EX vivo skin permeation of free TET and TET NE through excised mouse skin. Ex vivo skin penetration was evaluated with Franz cell (PermeGear V6-CA, US). Firstly, mouse skin was collected and covered on Franz cell with the dermal layer facing down. The receptor cell was filled with 8.0 mL of 20 % ethanol-water (v/v), and free TET and TET NE (2 mL, 1.5 mg/mL) were added in the donor cell. After that, the receptor cell and donor cell were clamped with a clip and connected to a water bath (32 °C). The effective diffusion area was 1 cm^2. After 24 h, the receptor phase was collected and detected with HPLC. The skin sample was rinsed with receptor phase for three times and cut into small pieces. The TET in the processed skin sample was extracted under ultrasonic for 30 min in 1 mL of methanol. After centrifugation at 12,500 g for 10 min, the TET concentration in the supernatant was measured with HPLC. The HPLC test was conducted with an Agilent 1200 Series HPLC system (Agilent, USA) equipped with a UV detector. An Agilent Zorbax SB-C18 column (250 mm × 4.6 mm, 5 μm, Agilent) was used to separate the sample at room temperature. The mobile phase was acetonitrile: methanol: water: acetic acid (40:30:30:1, v/v/v/v) was at a flow rate of 1 mL/min. The detected wavelength was set at 280 nm. The injection volume was 20 μL. Data were shown as means ± SD. $^{**}P < 0.01$, $^{***}P < 0.001$ compared with the indicated group.
